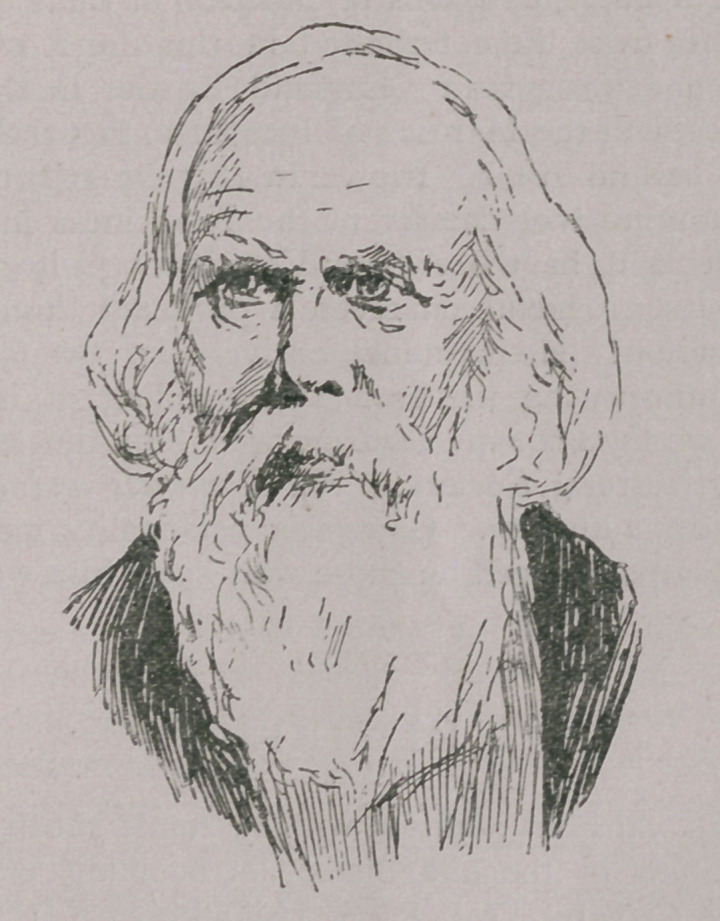# Joseph Gamgee

**Published:** 1895-05

**Authors:** 


### OBITUARY.

### JOSEPH GAMGEE.

Joseph Gamgee died at Edinburgh, Scotland, on February 9, 1895. Mr. Gamgee had been for over sixty years one of the well-known members of the veterinary profession, and for half a century he had been a celebrity. He was born in Essex, England, on the 10th of February, 1801. He lived an energetic life, and only failed in health recently, so that when his son died he was not allowed to hear of it. At the age of twenty Gamgee obtained a position under the Prince of Petrulla, in the south of Italy, to take charge of hunters and hounds. On his way he had his horses shod in Paris, and made comment on the superior farriery to be found in France. Gamgee's employment required frequent trips between Naples and Prince Petrulla's country estates, and brought him in contact with the brigands who invested the country, and he is credited with great bravery in several fights and escapes from them and from highway robbers.

Two years latter Gamgee returned to England and entered the Veterinary College at London. He afterward took a course at Alfort. In 1825 he returned to Naples, and shortly afterward removed to Florence, where he was joined by his wife and children from England. In Florence he gained a high reputation, having an extensive practice in the stables of the rich English who then travelled in their own coaches. In 1844 Gamgee was sent to England by the Duke of Savoy, in company with the Marquis of Pamparra, to purchase horses. He made several similar trips. In 1855 he returned to England, and successively taught in the New Veterinary College, Edinburgh, and in the Albert Veterinary College, London. Gamgee was a careful observer, a profound thinker, and an educated writer. He wrote extensively for the journals, and was an authority on the care and breeding of horses. His *Breeding of Hunters and Roadsters* won him the medal of the Royal Agricultural Society. His *Lameness and Horseshoeing* has been an authority. Gamgee's personality made him respected by all and loved by those who knew him well.

## Figures and Tables

**Figure f1:**